# Prevalence of enteric infections among hospitalized patients in two referral hospitals in Ghana

**DOI:** 10.1186/s13104-017-2621-x

**Published:** 2017-07-17

**Authors:** R. Akuffo, G. Armah, M. Clemens, K. C. Kronmann, A. H. Jones, P. Agbenohevi, K. Sagoe, N. Puplampu, N. Talla Nzussouo, W. Ampofo, K. Koram, C. Duplessis, E. Dueger

**Affiliations:** 1 Noguchi Memorial Institute for Medical Research, Accra, Ghana; 2 Global Disease Detection & Response Program (GDDRP), U.S. Naval Medical Research Unit No. 3 (NAMRU-3), Cairo, Egypt; 3 Global Disease Detection (GDD) Egypt Regional Center, U.S. Naval Medical Research Unit No. 3, NAMRU-3, PSC 452, P.O Box 5000, Foster city, FPO, AE 09835-9998 USA; 4 U.S. Naval Medical Research Unit No. 3, Ghana Detachment, Accra, Ghana; 5 37 Military Hospital, Accra, Ghana; 6 Tamale Teaching Hospital, Tamale, Ghana; 7 U.S. Centers for Disease Control and Prevention, Atlanta, GA USA; 8 Uniformed Services University of the Health Sciences, Bethesda, MD USA

**Keywords:** Prevalence, Enteric, Infections, Pathogens, Diarrhea, Surveillance, Hospitalized

## Abstract

**Background:**

Diarrhea is an important cause of morbidity and mortality worldwide. In Africa and Ghana in particular, it is estimated to contribute directly to 19 and 25% of pediatric mortality among children under 5 years, respectively.

**Methods:**

Surveillance for hospitalized acute diarrheal illness was initiated in November 2010 through October 2012 in a referral hospital in southern Ghana, and a teaching hospital in northern Ghana. Consenting hospitalized patients who met a standardized case definition for acute diarrheal illness provided demographic and epidemiologic data. Stool samples were collected and tested by culture for bacteria and by enzyme immunoassays for a panel of viruses and parasites.

**Results:**

A total of 429 patients were enrolled; 216 (50.3%) were under 5 years, and 221 (51.5%) were females. Stool samples were received from 153 patients. Culture isolates included *Shigella* sp., *Salmonella* spp., *Plesiomonas* sp. and *Vibrio cholerae*. Of 147 samples tested for viruses, 41 (27.9%) were positive for rotaviruses, 11 (7.5%) for astroviruses, 10 (6.8%) for noroviruses, and 8 (5.4%) for adenoviruses. Of 116 samples tested for parasitic infections; 4 (3.4%) were positive for *Cryptosporidium* sp. and 3 (2.6%) for *Giardia lamblia*. Of the enrolled patients, 78.8% had taken antibiotics prior to sample collection.

**Conclusions:**

Diarrheal pathogens were identified across all ages, however, predominantly (81%) in the children under 5 years of age. This study also detected high antibiotic use which has the potential of increasing antibiotic resistance. The most common enteric pathogen detected (49.4%) was rotavirus.

**Electronic supplementary material:**

The online version of this article (doi:10.1186/s13104-017-2621-x) contains supplementary material, which is available to authorized users.

## Background

Diarrheal disease, caused by a wide variety of viral, bacterial and parasitic pathogens, is a common cause of morbidity, hospitalization and mortality worldwide and occurs among people of all ages [1, 2]. It is the second leading cause of death among children under 5 years and is estimated to have caused 0.578 million pediatric deaths (0.448–0.750 million; 9.2%, 7.1–11.9) worldwide [3–6] with most deaths occurring in children in developing (low and middle income) countries. In Africa, diarrhea is estimated to cause 19% of childhood (<5 years) deaths [3] and accounts for 25% of pediatric mortality in Ghanaian children <5 years of age [7].

Some recent studies have attempted to describe the burden, epidemiology and etiological fraction of diarrhea in developing countries [8, 9] including Ghana [10, 11]. Most attributable cases of moderate-to-severe diarrhea identified in these studies included rotavirus, norovirus, astrovirus, *Cryptosporidium parvum*, *Campylobacter* spp., *Vibrio cholerae*, *Aeromonas* spp., enterotoxigenic *Escherichia coli* producing heat-stable toxin (ST-ETEC; with or without co-expression of heat-labile enterotoxin), and *Shigella* spp. [8–10]. While this development is encouraging, there is still limited data on diarrhea among hospitalized patients across all ages in Ghana.

In this work, we present results regarding the epidemiology and prevalence of acute diarrheal illness in Ghana based on a hospitalized study group.

## Methods

Surveillance for hospitalized acute diarrheal illness was initiated in November 2010 until October 2012, in two public referral hospitals in Ghana; 37 Military Hospital (37MH) in the Greater Accra Region (Southern Ghana), and the Tamale Teaching Hospital (TTH) in the Northern Region of Ghana (Additional file 1).

A case of hospitalized acute diarrheal illness was defined as a hospitalized adult or child aged 31 days or older with the passage of three or more watery or loose stools in a 24-h period or more but less than 14 days in duration. This criteria also included hospitalized patients aged 31 days and above with two loose or liquid stools in a 24-h period or more, prior to admission and at least one of the following symptoms: history of fever (with current illness), dysentery, abdominal pains/cramps, nausea and vomiting, within a 14 day period. Suspected cases were identified from the admissions log book on the infectious disease wards of participating hospitals based on chief complaint of diarrhea reported at the time of admission. Hospitalized patients who met the case definition for diarrheal illness or their parents or legal guardians (in the case of children) were invited to participate in the study. Patients who developed diarrhea after admission were not included in the study.

Demographic and epidemiological data were obtained by the administration of a structured questionnaire using personal digital assistants (PDAs). Data was also collected on antibiotic use prior to hospital admission and prior to sample collection. Hospital folders of consented patients were also monitored for outcome information including the weekly monitoring of antibiotic administration (if any) after specimen collection.

Stool samples were collected from enrolled patients within 24 h of admission and stored at 2–8 °C at the study sites and transported within 24 h of sample collection to NMIMR at same temperature, using cool box with ice packs (without stool transport medium such as Cary Blair) for stool culture as well as virus and parasite enzyme immunoassay (EIA) testing respectively. Samples for bacteria culture were plated on MacConkey, Salmonella/Shigella and TCBS agar plates and cultured for 24–48 h at 37 °C. Apart from *Vibrio cholerae,* isolates were identified using biochemical methods and confirmed with the API-20E bacterial identification test strip from BioMerieuxInc©, France. Antimicrobial susceptibility testing (AST) was performed on isolates using disc diffusion method, with the cut-off inhibition zone diameter values for resistance, intermediate and susceptible determined using the performance standards for antimicrobial susceptibility testing twenty-first informational supplement [12].

AST to trimethoprim/sulphamethoxazole, ampicillin, ciprofloxacin, amikacin, gentamycin, cephalothin, ampicillin-sulbactam, nalidixic acid, imipenem, cefotaxime, chloramphenicol, tetracycline, and aztreonam was conducted for *Salmonella* sp. and *Plesiomonas* sp. respectively. Wampole™ Giardia II, *E. histolytica* and *Cryptosporidium* test kits, from Techlabs, USA, were used for the parasite enzyme immunoassays (EIA). The IDEIA ™Rotavirus, Adenovirus, Norovirus and Astrovirus test kits (OxoidLtd, UK) were used for the virus EIAs. If a sample was insufficient for all testing, lab tests were prioritized in the following order: stool culture, viral EIA and parasite EIA, respectively.

All statistical tests were conducted at a 95% confidence level.

## Results

A total of 429 hospitalized patients who met the case definition for acute diarrheal illness provided consent and assent (for children) for study inclusion (Table 1). Age range of enrolled patients was 1 month to 95 years; 216 (50.3%) were under 5 years, and 221 (51.5%) were females (Table 2). Symptoms reported by participants include vomiting, history of fever, abdominal cramps, nausea and dysentery in 347 (80.9%), 300 (69.9%), 156 (36.4%), 78 (18.2%) and 57 (13.3%).

**Table 1:** Distribution of enrolled participants and samples received from study sites

Study site	Enrolled, n (%)	Samples received, n (%)
TTH	141 (32.9)	44 (28.8)
37MH	288 (67.1)	109 (71.2)
Total	429	153

*TTH* Tamale Teaching Hospital, *37MH* 37 Military Hospital

**Table 2:** Demographic and clinical characteristics of enrolled ADI cases

Characteristic	Subgroups	Enrolled (N = 429)
Age group	31 days to <1 years	98 (22.8)
	1 to <5 years	118 (27.5)
	5 to <18 years	21 (4.9)
	18 to <65 years	171 (39.9)
	65 years+	21 (4.9)
	Male	200 (46.6)
Sex	Female	221 (51.5)
	Missing	8 (1.9)
	Antibiotic use prior to admission	49 (11.4)
Antibiotic use	Antibiotic use prior to sample collection	338 (78.8)
Outcome^a^	Discharge	395 (96.6)
	Death	12 (2.9%)
	Unknown/missing	22 (5.4)

Data is represented in N (%)

^a^Outcome data available for 409 participants

Duration of diarrheal symptoms experienced by the enrolled participants is presented in Table 3, of which majority (90.2%) were within 0–5 days. In addition, majority of the enrolled patients 232 (54.0%) were hospitalized for a duration of 1–3 days while 110 (25.7%), 37 (8.7%), and 50 (11.6%) patients were hospitalized for periods of 4–6 days, 7–9 days and >10 days respectively.

**Table 3:** Duration of diarrheal symptoms among enrolled study participants (N = 429); Nov 2010–Sep 2012

Duration of diarrheal symptoms (days)	Number of participants
0–1	98 (22.8)
2–3	185 (43.2)
4–5	104 (24.2)
6–7	35 (8.2)
8–9	3 (0.7)
10–12	4 (0.9)

Data is represented in N (%)

Forty-nine (11.4%) of the enrolled cases reported antibiotic use prior to hospital admission; the indication for antibiotic use prior to hospital admission was however unclear. Furthermore, antibiotics, including ciprofloxacin (41.5%), metronidazole (22.8%), cefuroxime (17.8%) and ceftriaxone (16.0%), were administered to 338 (78.8%) of the consented patients prior to specimen collection.

Data on outcome of hospitalization was available for 409 (95.3%) of the enrolled patients; 395 (96.6%) were discharged, and 12 (2.9%) died. Of the patients who died, 5 (41.7%), 3 (25%), 3 (25%), and 1 (9.1%) had outcome diagnoses (the diagnosis given to the participant closest in time to their death) of gastroenteritis, pneumonia, malaria, and congestive cardiac failure respectively. The mean age of the patients who died was 19 years (range 5 months to 52 years). Four (33.3%) of those who died submitted stool samples for processing. Of these, three tested positive for specific enteric pathogens; rotavirus [2] and adenoviruses [1].

A total of 153 (35.7%) stool samples (Table 1) were collected from the enrolled patients, of which 99 (64.7%) were from children under 5 years. All 153 samples were cultured for bacterial isolates. Viral EIA was performed on 147 (96.1%) of the samples; six of the samples had insufficient quantity after culture. Parasite EIA was conducted on 116 (75.8%) samples; the remaining had insufficient quantity of sample following viral EIA testing.

Bacterial pathogens were isolated from 6 (3.9%) of the 153 stool samples cultured. These included one *Shigella* spp., one *Vibrio cholerae*, two *Salmonella* sp., and two *Plesiomonas shigelloides*. Although *Escherichia coli* was identified in 62 (40.5%) of the samples tested, this study did not determine whether the *E. coli* observed were diarrheagenic.

The *Salmonella* sp. isolates were resistant to nalidixic acid, chloramphenicol, tetracycline, ampicillin, ciprofloxacin, and aztreonam antibiotics while the *Plesiomonas* sp. were resistant to trimethoprim/sulphamethoxazole, ampicillin, amikacin, gentamycin, cephalothin, nalidixic acid, imipenem, tetracycline and aztreonam (Table 4). ASTs were not conducted for the *Vibrio cholerae* and *Shigella* sp.

**Table 4:** Antimicrobial susceptibility testing results for *Salmonella* sp. and *Plesiomonas* sp.

Antimicrobial agent	Disk conten (μg)	Inhibition zone diameter for different pathogens (mm)^b^			
		*Salmonella* sp. 1	*Salmonella* sp. 2	*Plesiomonas* sp. 1	*Plesiomonas* sp. 2
Amikacin	30	18	17	12^a^	12^a^
Ampicillin	10	8^a^	9^a^	8^a^	8^a^
Ampicillin–sulbactam	10/10	18	18	17	19
Aztreonam	30	14^a^	12^a^	13^a^	14^a^
Cefotaxime	30	28	27	30	29
Cephalothin	30	20	21	11^a^	12^a^
Chloramphenicol	30	9^a^	9^a^	15	16
Ciprofloxacin	5	12^a^	11^a^	24	23
Gentamycin	10	14	13	8^a^	8^a^
Imipenem	10	26	24	17^a^	15^a^
Nalidixic acid	30	10^a^	9^a^	9^a^	8^a^
Tetracycline	30	8^a^	8^a^	10^a^	9^a^
Trimethoprim/sulfamethoxazole	23.75	18	19	8^a^	8^a^

^a^Resistant

^b^Cut-off inhibition zone diameter values determined using the performance standards for antimicrobial susceptibility testing twenty-first informational supplement for CLSI [12]

**Fig. 1:**
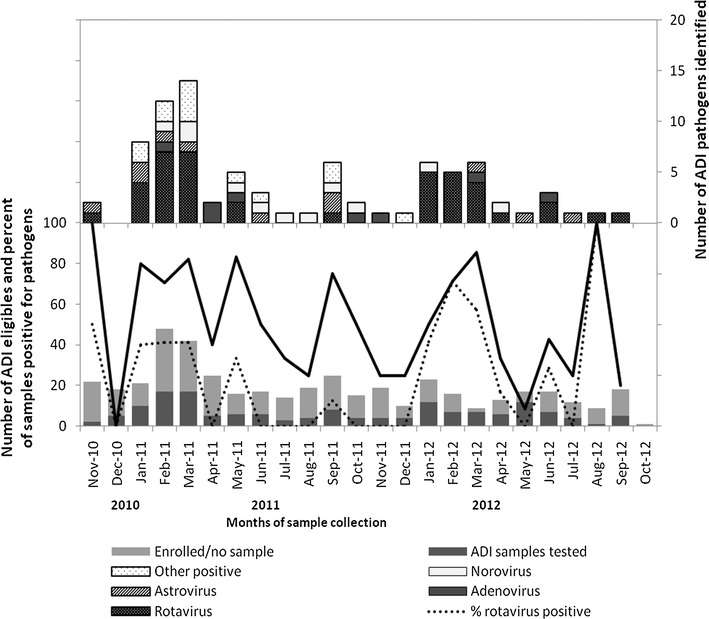
Monthly distribution of ADI enrolled cases (*lower panel*, n = 429) and pathogens detected from samples tested (*upper panel*, n = 83); Nov 2010–Oct 2012

Of the 147 samples tested by viral EIA, 41 (27.9%) were positive for rotaviruses, 11 (7.5%) for astroviruses, 10 (6.8%) for noroviruses and 8 (5.4%) for adenoviruses. Two of the rotavirus positive samples also tested positive for noroviruses while one sample was positive for both astrovirus and norovirus. In addition, one of the rotavirus positive samples was also positive by culture to *Shigella* sp. and *Plesiomonas shigelliodes*. Furthermore, our study identified peaks in rotavirus infection during January–March of the years 2011 and 2012 respectively (Fig. 1). Of the 116 samples tested for parasitic infections; 4 (3.4%) were positive for *Cryptosporidium* sp. and 3 (2.6%) for *Giardia lamblia* respectively. The majority (81%) of enteric pathogens detected were among children under 5 years (Table 5). No significant difference was observed between the enrolled participants who provided sample and those who did not provide sample.

**Table 5:** Age distribution of stool pathogens detected (N = 83); Nov 2010–Sep 2012

Stool pathogens	Frequency	Age group^a^		
		31 days to <1 years	1–5 years	>5 years
Rotavirus	41	17 (41.5)	22 (53.7)	2 (4.9)
Norovirus	10	3 (30.0)	2 (20.0)	5 (50.0)
Astrovirus	11	4 (36.4)	2 (18.2)	5 (45.5)
Adenovirus	8	4 (50.0)	3 (37.5)	1 (12.5)
*Cryptosporidium*	4	3 (75.0)	0 (0)	1 (25.0)
*Giardia*	3	2 (66.7)	0 (0)	1 (33.3)
*Salmonella* sp.	2	1 (50.0)	1 (50.0)	0 (0)
*Plesiomonas* sp.	2	2 (100.0)	0 (0)	0 (0)
*Vibrio Cholerae*	1	0 (0)	0 (0)	1 (100.0)
*Shigella* sp.	1	1 (100.0)	0 (0)	0 (0)
Total	83	37 (44.6)	30 (36.1)	16 (19.3)

^a^Data is represented in N (%)

## Discussion

Most (84.3%) of the enteric pathogens identified in our study were viruses of which rotavirus constituted the majority (58.6%) (Table 5). Rotavirus is known to be the most common cause of severe acute, watery diarrhea in children under 5 years of age in industrialized and developing parts of the world [13], with over 80% of deaths attributable to it, and occurring in the poorest nations of South Asia and sub-Saharan Africa [14, 15].

Multiple studies in Ghana among children <5 years of age confirm the prominent role of rotavirus in pediatric gastroenteritis [10, 11, 16–19]. Our study identified peaks in rotavirus infection during January–March of the years 2011 and 2012 respectively (Fig. 1) while earlier diarrhea surveillance studies in other regions of Ghana identified diarrhea peaks in October–March [22–25].

Two rotavirus vaccines are available for use and are recommended by the World Health Organization (WHO) for inclusion in the routine immunization program of nations [20] and has been part of Ghana’s expanded program of immunization (EPI) since late April 2012 [21].

Recent studies in Ghana have reported decline in hospitalization due to severe diarrhea after the introduction of the rotavirus vaccines and we anticipate that ongoing surveillance for rotavirus disease in Ghana will reveal a further decrease in the absolute prevalence of rotavirus associated diarrheal disease and hospitalization [21].

Although almost 50% of samples tested positive for pathogenic viruses in this study, the percentage of cases with identifiable bacterial and parasitic pathogens (~4%) was relatively small compared to other studies which recorded up to 20–30% identifiable bacterial and parasitic pathogens [4, 7, 26–31]. However, studies on the etiologies of acute diarrhea in non-hospitalized patients in Ghana unveiled a 5% prevalence rate of bacterial and parasitic pathogens [19] very similar to what we have observed in this study. The overall low bacterial pathogen detection rate in our study is likely multi-factorial.

With the exception of suspected Shigella (dysentery) and cholera, the WHO strongly discourages the use of antibiotics in the treatment of diarrhea since indiscriminate antibiotic use increases resistance to antibiotics of many disease-causing organisms [32, 33]. Previous studies in Ghana have reported the emergence of antibiotic resistance of diarrheal pathogens [34, 35]. As a result, the high use of antibiotics for treatment of diarrhea in Ghana should be of concern to all (Table 5).

*Giardia lamblia*, *Entamoeba histolytica*, and *Cryptosporidium parvum* are the major parasitic organisms causing childhood diarrhea in developing countries [28]. These have been estimated to be associated with 15–20 and 2–5% of diarrhea cases in developing and developed countries respectively [30]. We identified Giardia and *Cryptosporidium* in 2.6 and 3.4% of participants respectively. The observed detection rates in our study was however lower than the 11.4% intestinal parasites incidence reported by Nkrumah [30] with Giardia being the most common (89.0%), and the 8.2% recorded for Cryptosporidium in children <2 years by Opintan et al. in Ghana [35].

Nkrumah et al. used direct microscopy of smears in saline to identify parasites while Opintan et al. used polymerase chain reaction to identify the Cryptosporidium. Both studies included outpatients and were focused on children under 5 years whereas our study focused on inpatients and included patients across all ages. The widespread antibiotic use encompassing metronidazole, prior to sample collection, may have contributed to the low parasite yield observed in our study.

Recent data on diarrhea in developing countries call for a paradigm shift for future studies on diarrhea in these countries, including Ghana. While some studies have previously suggested that certain diarrheal pathogens such as enterotoxigenic Escherichia coli and rotaviruses predominate in developing countries, with others being more common globally and in developed areas [36], available data suggests that there is no evidence that any particular pathogen or type of pathogen is associated with persistent diarrhea in children under the age of six in low and middle income countries [4]. There is therefore a need for future diarrheal surveillance in developing countries and Ghana in particular, to ensure that a wide range of diarrheagenic pathogens are tested.

## Conclusions

Diarrheal pathogens identified among hospitalized patients in the Greater Accra and Northern regions of Ghana occurred across all ages and included bacteria, parasites and viruses. In view of the high antibiotic use observed in this study, antibiotic susceptibility testing should be integrated into surveillance programs to dictate the appropriate antibiograms which are always in flux.

## Limitations

- This study was not population based, so only represents infection prevalence among hospitalized cases who provided stool samples, not burden of diseases in general population.
- A large proportion of enrolled cases did not provide samples mainly because they were discharged within a day after admission, before the surveillance team could obtain sample from them. This may have biased available data on duration of hospitalization.
- Stool transport medium (such as Cary Blair) was not used and hence may have likely contributed to the low bacterial isolation detected.
- This study did not look for *Campylobacter* and diarrheagenic *E. coli* in the samples collected.
- The high antibiotic use observed in this study may have had an effect on the bacterial yield observed.

## Additional file

**Additional file 1.** Study site information.

## Abbreviations

ADI: acute diarrheal infections
EIA: enzyme immunoassay
GHS: The Ghana Health Service
NAMRU-3: US Naval Medical Research Unit No. 3
CDC: US Centers for Disease Control and Prevention
WHO: World Health Organization
EPI: expanded program of immunization
