# Supplementary material for: Prevalence of enteric infections among hospitalized patients in two referral hospitals in Ghana
Source: BMC Res Notes. 2017 Jul 17;10:292. doi: 10.1186/s13104-017-2621-x (PMC5514524; doi:10.1186/s13104-017-2621-x)
Supplement: Supplementary file 1 — Additional file 1. Study site information. [file 13104_2017_2621_MOESM1_ESM.docx]

Study Site Information

The 37 Military Hospital is a specialist hospital located in Accra and is the second largest hospital in Ghana after the Korlebu Teaching hospital. Although the 37 Military Hospital is staffed primarily by military personnel, it provides services to both the military and the general public. The hospital has about 400 beds and provides 24-hour medical services. Currently, the hospital also serves as a teaching hospital for post-graduate medical students.

The Tamale teaching hospital is located in the eastern part of the Tamale metropolis in the Northern Region of Ghana and serves as a medical referral centre for the Northern, Upper East and Upper West Regions, and the northern parts of the Brong Ahafo Region of Ghana. The hospital has a bed capacity of about 340 and serves as a clinical teaching institution to the School of Medicine and Health Sciences, University for Development Studies (SMHS-UDS) in Tamale and the Ghana College of Physicians and Surgeons.
